# Recent surgical options for vestibular vertigo

**DOI:** 10.3205/cto000140

**Published:** 2017-12-18

**Authors:** Stefan Volkenstein, Stefan Dazert

**Affiliations:** 1 Department of Otolaryngology, Head & Neck Surgery, Ruhr-University of Bochum at the St. Elisabeth Hospital of Bochum, Germany

**Keywords:** peripheral vestibular vertigo, surgical treatment, Menière’s disease, dehiscence syndrome, endolymphatic sac surgery, vestibular implants

## Abstract

Vertigo is not a well-defined disease but a symptom that can occur in heterogeneous entities diagnosed and treated mainly by otolaryngologists, neurologists, internal medicine, and primary care physicians. Most vertigo syndromes have a good prognosis and management is predominantly conservative, whereas the need for surgical therapy is rare, but for a subset of patients often the only remaining option. In this paper, we describe and discuss different surgical therapy options for hydropic inner ear diseases, Menière’s disease, dehiscence syndromes, perilymph fistulas, and benign paroxysmal positional vertigo. At the end, we shortly introduce the most recent developments in regard to vestibular implants. Surgical therapy is still indicated for vestibular disease in selected patients nowadays when conservative options did not reduce symptoms and patients are still suffering. Success depends on the correct diagnosis and choosing among different procedures the ones going along with an adequate patient selection. With regard to the invasiveness and the possible risks due to surgery, in depth individual counseling is absolutely necessary. Ablative and destructive surgical procedures usually achieve a successful vertigo control, but are associated with a high risk for hearing loss. Therefore, residual hearing has to be included in the decision making process for surgical therapy.

## 1 Introduction

The surgical treatment of different diseases of the peripheral vestibular system is one of the most controversially discussed topics within the otolaryngological scientific society as well as with neighbouring disciplines. According to the current literature, a surgical intervention is indicated in slightly more than 1% of the patients at hospitals specialized for vertigo [1]. In selected disease such as MM about 20% of the patients may need surgical therapy in the course of the disease [2], [3], [4], [5]. Retrospectively, certain interventions or diseases are particularly in the focus of the discussion. In the 1960ies and 1970ies the “spontaneous perilymph fistula” was mainly in the USA controversially discussed, in the 1980ies, it was the surgery of the endolymphatic sac, and since 2000, it is the dehiscence syndrome of the superior semicircular canal. The available literature for studies of distinct surgical procedures is often very heterogeneous. Because of this the surgery of the endolymphatic sac and also the selective transection of the vestibular nerve are defined as obsolete in the (not yet revised) guidelines on “vertigo” of the German Society of Neurology and by other authors of the neurological discipline [6], [7]. Beside a certain diagnostic vagueness of some of the diseases that will be discussed in the following, another reason might possibly be the heterogeneity of the investigated and compared patient groups and the not clearly defined success rates of the respective procedures in single trials (e.g. improvement vs. complete elimination of the symptoms). A Cochrane review is only available for saccus surgery and this is based on the results of 2 studies with only 59 patients [8], [9]. Observational trials with many thousands of operated patients were not included because they did not meet the strict quality criteria of the Cochrane Collaboration. For all other surgical therapy modalities, there is a lack of randomized placebo-controlled studies. 

The development of additional functional diagnostic procedures in neuro-otology over the last decades allows a more exact diagnosis and differentiation of distinct diseases [10] and is associated with a revised indication for surgical interventions for diseases of the peripheral vestibular system raising hope for a more targeted application of invasive and partly destructive therapeutic modalities [11].

Since the diagnostics of the peripheral vestibular system is the topic of another contribution, it will not be in the focus here. Due to the restricted capacity of this booklet, the etiology of each disease will only be described when it is highly relevant for therapy. In the following, the development of the surgical procedures will be summarized for each entity and discussed based on current trials in order to be able to give a recommendation as a possible therapeutic option after failure of alternative procedures.

## 2 Hydropic inner ear diseases – Menière’s disease and Menière’s syndrome

### 2.1 Overview and terminology

The paroxysmal occurrence of the classic symptom triad of rotational vertigo, fluctuating hearing ability, and tinnitus is called Menière’s disease or Morbus Menière (MM) according to Prosper Menière (1861) [12]. Later, further symptoms (sense of pressure/fullness in the ear, nausea and vomiting, falls etc.) were described in the context of this inner ear or labyrinthine disease. Already in 1938, 2 teams succeeded independent from each other in allotting the endolymphatic hydrops (ELH) as pathological correlate for this disease [13], [14]. Since then, there are several disease entities with a manifold and partly confusing terminology that are associated with ELH: cochlear and vestibular Menière’s disease, monosymptomatic Menière’s disease etc. [4], [15], [16], [17]. Even if ELH is known to be the cause for the symptoms of MM for a long time, guidelines still recommend diagnosis based on medical history, audiometry, and the principle of exclusion diagnostics [17], [18], [19], [20], [21], [22]. In the future, the possibility of visualization of ELH by means of magnet resonance imaging in living patients [23] will contribute to easier exclude other diseases such as for example vestibular migraine that appears with similar symptoms [24]. This development contributes to an improved and more specific diagnosis of hydropic inner ear diseases and will probably lead to a revision of the mentioned clinical and audiological diagnostic criteria [25]. According to the most recent classification, the term of hydropic inner ear diseases summarizes different diseases that have an endolymphatic hydrops as morphological correlate. They are subdivided into primary and secondary hydropic inner ear diseases [26]. In some articles, the term of Menière’s syndrome is used. In the context of Menière’s syndrome, the symptoms appear secondarily because of a known primary disease, e.g. a pressure dysequillibrium in the middle ear due to Eustachian tube dysfunction or pathologies of the ossicular chain may influence the inner ear pressure [27] or tumors and inflammatory diseases may cause the ELH [17], [28]. In contrast to Menière’s syndrome, the ELH in MM develops idiopathically because of absorption disorders and/or deregulated production of endolymph fluid without known pathologies that lead to a pathologically changed endolymph homeostasis [13], [29].

### 2.2 Surgical procedures

**Figure 1:**
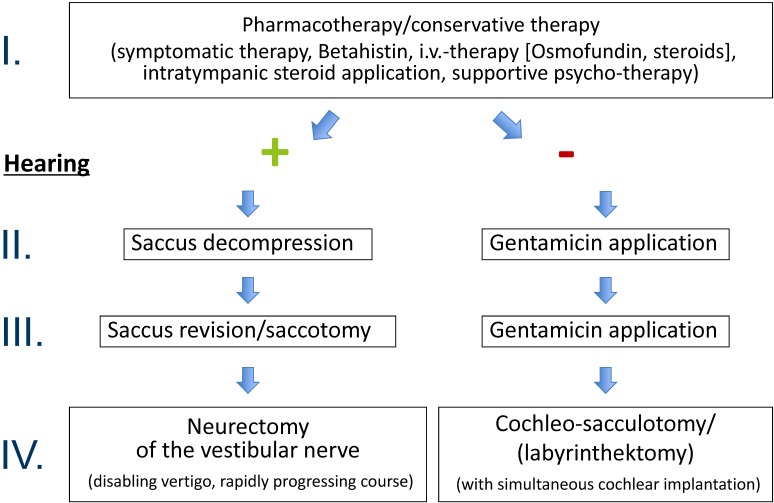
Bochum step-wise therapeutic scheme for patients with Menière’s disease

**Figure 2:**
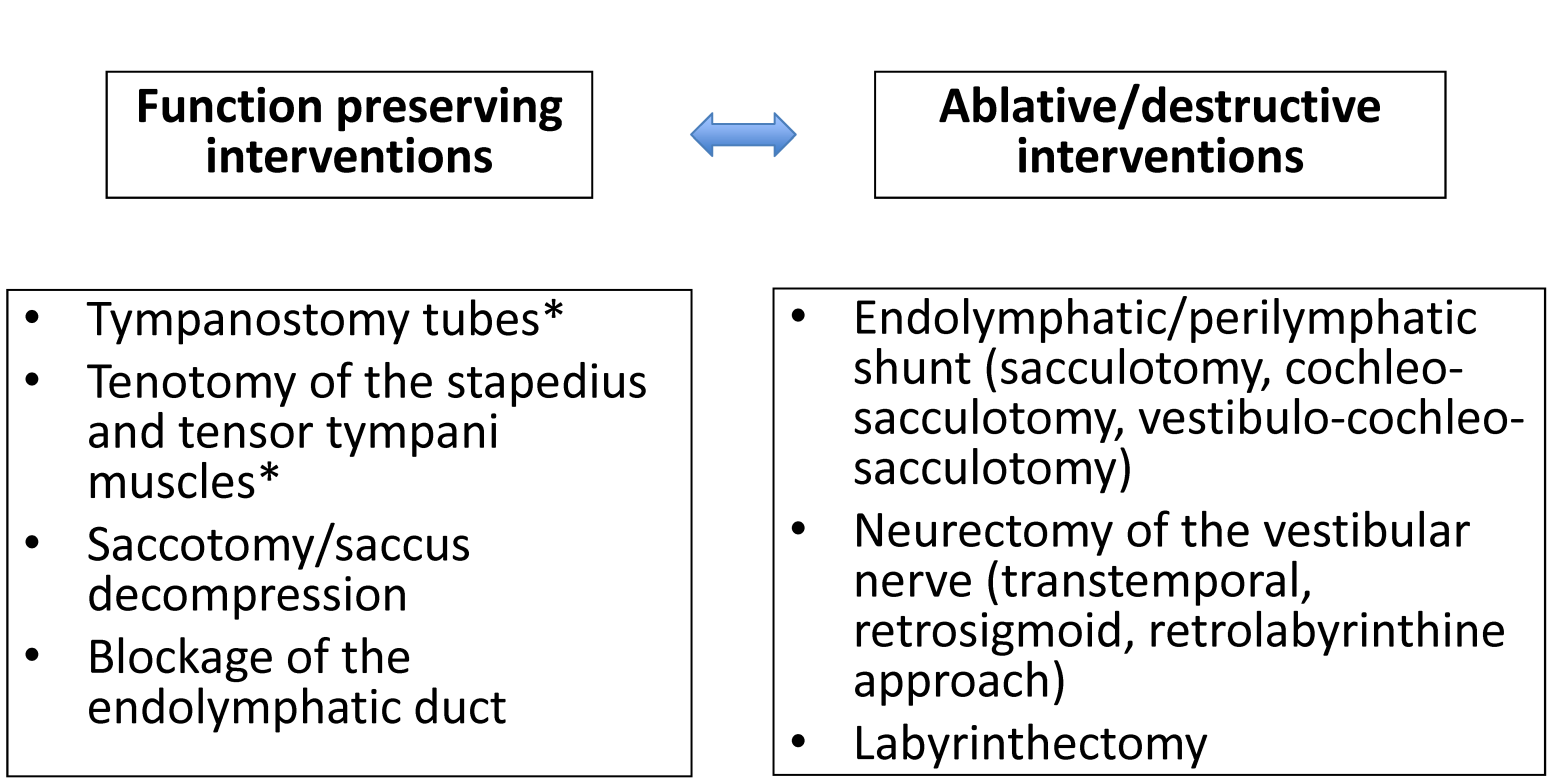
Surgical treatment procedures for patients with hydropic inner ear disease (* especially patients with Menière’s syndrome and pathological pressure conditions in the middle ear)

Therapy of MM is primarily conservative and different gradual schemes are available that may be applied depending on the progress of the disease and the residual hearing ability [4], [30], [31], [32] (Figure 1 (Fig. 1)). After failure of conservative therapeutic approaches, partly even despite application of off-label pharmaceutics, repeatedly verification of the diagnosis, and persisting symptom attacks indicate surgical therapy when the disease can be clearly assigned to one side. The indication prevalence for surgical interventions is different in the course of the disease and varies between 1 and 25% of the patients suffering from MM [2], [3], [6], [33], [34], [35], while the percentage is decreasing since the administration of intratympanic corticosteroids and gentamicin becomes more popular as well [36]. Not only in the context of treating patients with MM, but in general, evidence plays an increasing role in the medical field. Meanwhile the number of randomized, placebo-controlled trials has tripled during the last two decades (from 1994 to 2013) with an increasing attention that is paid to standardized assessment of the quality of life as additional evaluation criterion for the success of a therapy [36]. Regarding the surgical procedures, there are function preserving and destructive options (Figure 2 (Fig. 2)) that can be performed with or without hearing preservation. In the following, these options will be described in more detail.

#### 2.2.1 Function preserving procedures

##### 2.2.1.1 Middle ear drainage

After Tumarkin had promoted tympanostomy tubes as possible therapeutic option for patients with MM in 1966 [37], a series of experimental and clinical investigations was performed in the following years that justified this procedure and in particular showed an improvement of the vestibular symptoms by tympanostomy tube insertion [38], [39], [40], [41], [42], [43]. The hearing ability was not changed significantly [44]. In the 1970ies and also later, critics rejected this method as being useless with the argument that not all MM patients suffered from Eustachian tube dysfunction [45], [46], [47]. Even when an investigation performed in 1988 by Montandon et al. [39] finally confirmed the positive results of earlier, not really systematic publications on this topic, there are still doubts regarding the benefit and the usefulness of tympanostomy tubes for MM. The major part of the effect is interpreted as placebo effect by several people and it was not possible to identify a clinical correlation between cochleo-vestibular disorders and tube dysfunctions [15], [48]. Thomsen et al. compared the results after tympanostomy tube insertion and after saccus decompression/saccotomy and could not confirm significant differences between both therapy methods, however, a reduction of the vertigo attacks after both interventions was observed so that in conclusion the less invasive tympanostomy tube insertion was suggested as first option after failure of pharmaceutic/conservative therapy [49]. Since it could be shown that pressure changes in the middle ear may lead to pressure changes in the inner ear, the influence on the symptoms of MM was explained by this fact in the following [43], [50]. Furthermore, Kimura and Hutta could show in an animal model that pressure changes in the middle ear could significantly influence an experimentally induced ELH [42]. Park et al. investigated the functional impact resulting from the insertion of tympanostomy tubes on the vestibular system and limited their trial to patients with MM who had a tube dysfunction (middle ear pressure <–50 daPa) [43]. Tympanostomy tubes did not lead to relevant complications and in 68.2% of the patients, the symptoms improved after tympanostomy tube insertion, however, without postoperative change of the sacculus or lateral semicircular canal function. The mechanisms that are responsible for the improvement of the complaints remain still unclear. It is important to select appropriate patients who might benefit from this therapy [28]. In this context, patients who have tube dysfunction with pathological middle ear pressure are recommended for tympanostomy tube insertion in selected cases [43]. The fact that some studies could not confirm the positive effects that are achieved by tympanostomy tubes, is possibly also based on the selection of inappropriate patients for this type of therapy [43]. Apparently there are patients who do not experience improvement after tympanostomy tube insertion [40], [44], [51]. Interestingly, some patients who benefited from tympanostomy tubes, had again vertigo attacks after extrusion of the tubes [39], [44] that disappeared again after tube re-insertion.

The tympanostomy tube insertion can be suggested for MM, especially in elderly patients, as early therapeutic option for a selected group of patients (e.g. with tube dysfunction) before applying an invasive or even destructive therapy procedure [44].

##### 2.2.1.2 Tenotomy of the tendons of the stapedius and the tensor tympani muscles

Tenotomy of the tendons of the tensor tympani muscle and/or the stapedius muscle are considered as possible function preserving therapy options in patients with MM that might positively influence the audiological symptoms beside the vestibular ones [27], [52]. This method that is mainly applied in Austria consists of transecting the tendons of both middle ear muscles to influence the pressure coupling of the ossicular chain to the perilymphatic space [27]. Beside the reduction of the incidence of vertigo attacks and their intensity, case series of 30 and 45 patients showed an improvement of the audiometric results, especially in the frequency range between 500 Hz and 3 kHz [52], [53]. In a subsequent investigation of 42 patients with a follow-up time of up to 9 years, Loader et al. reported a significant reduction of the dizziness handicap inventory (DHI), a questionnaire on the subjective self-assessment of the impairment by vertigo complaints. It could also be shown that the therapeutic success was higher, the more severe the preoperative impairment was estimated [54].

In a retrospective study, Albu et al. investigated the results between tenotomy and endolymphatic mastoid shunt surgery in a homogeneous patient group [55]. While there were no differences regarding tinnitus, the symptom control of fullness sensation, vestibular symptoms, and especially hearing ability were significantly better in the group of patients with tenotomy; additionally 25% of the patients had to undergo revision after shunt surgery.

In a comparative study between an intratympanic gentamicin therapy alone and in combination with the described tenotomy, it could be revealed that there is no additional benefit after tenotomy regarding the quality of life, DHI, tinnitus, or the incidence of the severity of vertigo attacks [56].

All trials have been performed in patients who suffered from definitive unilateral MM according to the AAO-HNS criteria. Equally to the tympanostomy tube insertion attention must be paid to the appropriate patient selection (see above) in order to achieve maybe even better results in the future [27].

##### 2.2.1.3 Saccotomy/saccus decompression

Even if decompression of the endolymphatic sac was the most frequently performed surgical intervention for MM for a long time [57], and is at least still in the USA today [58], surgery involving those anatomical structures remain the most controversially discussed procedures [27], [59], [60]. In 1927, Portmann was the first to describe an intervention of the endolymphatic sac for treatment of vertigo [61]. Since then several interventions at the endolymphatic sac were reported [57], [62], [63], [64], [65], [66], [67]: only decompression of the endolymphatic sac, only incision (called saccotomy or endolymphatic shunt surgery), and the incision with insertion of silicone or Teflon sheets [68], [69]. Yokuta et al. suggest in addition to saccotomy the intraoperative administration of corticosteroids directly into the endolymphatic sac [5]. Other procedures such as shunt surgery between the endolymphatic sac and the subarachnoidal space [70] could not prevail. Despite the success rates of saccus surgery mentioned in the literature amounting to 60–90% [57], [67], [71], [72], [73], [74], [75], [76], [77], [78], [79], [80], these interventions are partly suspected to have unspecific or placebo effects [8], [9], [49], [81], [82], [83]. Interestingly, a post-mortem study of patients having undergone interventions at the endolymphatic sac could not reveal a clear correlation between the complaints and the surgical outcome so that some patients had experienced improvement of the complaints even if the target structure had not been reached in the context of surgery. Other patients who definitely had surgery of the endolymphatic sac, did not show an improvement of the preoperative stage [84].

The interventions at the endolymphatic sac were especially in the focus of the critics because of an article published by Thomsen et al. in 1981. In this large randomized, placebo-controlled trial, no difference could be confirmed between saccotomy and mastoidectomy without exposition of the endolymphatic sac [82]. Follow-up studies to this publication were added after 3 and after 9 years [85], [86] which allow similar conclusions, i.e. that there is no difference between the actual (saccotomy) and the placebo intervention. Since the publication of the original paper in 1981, there are doubts if the conclusions of the presented data were justified [87], [88]. In 1983, Pilsbury investigated the data again and revealed a significantly better vertigo control by saccotomy in contrast to placebo surgery [89]. Welling analyzed the original data of the study of 1981 again and demonstrated that there is a statistically significant improvement in the saccotomy group with regard to specific aspects (especially vertigo, tinnitus etc.) [90]. A Cochrane review of 2010 about the surgical options for patients with MM was again the topic of further discussions around saccus surgery, also in its revised version of 2013, because no sufficient evidence for the benefit of saccus surgery in patients with MM could be supported by valid data as their conclusion [8], [9]. Due to the strict inclusion criteria, the assessment is based on only 2 articles with a total of 59 patients. The already mentioned 9-years follow-up by Bretlau et al. regarding the study of Thomsen (1981) and another study from the same group comparing saccotomy with the results of tympanostomy tube insertion as placebo intervention in a non-blinded, randomized study [49]. In both trials no statistically significant difference in the response rate of saccotomy and the placebo intervention could be revealed although the overall symptoms improved in about 70% of the patients. This was considered as unspecific effect of all surgical interventions. Other studies that were performed in a relevantly larger patient population, but lacked the criteria of the Cochrane Collaboration, were ignored by the critics. 

In 1988, Monsell et al. reported about a study of 83 patients with a success rate of 75% [91]. In 2002, Paparella and Fina published a series of more than 2000 interventions at the endolymphatic sac and achieved a complete control of the vertigo symptoms in 75% and an improvement in 90% of the treated patients with a hearing preservation rate of 98% [92]. The authors emphasized particularly their surgical technique including a complete mastoidectomy with broad decompression of the sigmoid sinus. Recurrent symptoms after improvement of the complaints occurred in 5% of the cases, typically 3–4 years after the first intervention. Huang reported about his summarized experiences with saccus surgery in 3000 cases and a success rate regarding vertigo symptoms of more than 90% after 2–3 years [93]. Lee et al. reported about satisfactory vertigo control in 78% of the patients in a series of 486 saccotomy interventions [94].

In 2014, Sood et al. compared the different interventions at the endolymphatic sac in a systematic review and meta-analysis [95]. The literature review addressed the problem with definition of the success rate of the different articles which made a comparison rather difficult. Although continuously revised guidelines exist [18], [19], [20], [21], they were not correctly applied in many of the cases [95]. Nearly 80% of the authors stated that they worked based on those guidelines, only half of them actually applied them correctly [96]. After evaluation of 36 trials, the conclusion could be drawn that control of the vertigo symptoms could be achieved in 75% of the patients after 12 months and after 2 years without a significant difference between saccus decompression and saccotomy/shunt surgery with regard to hearing preservation and control of the vertigo symptoms. Only the evaluation of the patients with saccotomy who underwent insertion of a silicone sheet or something similar, had a significantly poorer hearing preservation, but no difference regarding the vertigo symptoms was observed so that the usefulness of sheet insertion is in doubt due to the missing benefit [95].

Yokota et al. reported about 263 patients who had the indication of saccotomy, but 56 decided against this intervention and received the best possible pharmaceutical/conservative therapy [5]. After 2 years success rates of 92.8% compared to 46.4% in the conservative group were reported. After 7 years, the success rate was 81.0% in the surgical group compared to 30% in the control group. Additionally, it could be shown that patients with a concomitant neurosis or depression had significantly lower success rates after surgical therapy than patients without those concomitant diseases. One of the conclusions is the recommendation to perform supportive psychological therapy in order to further improve the surgical outcome as well as the conservative results.

If an intervention at the endolymphatic sac was successful at first (at least one year of improved symptoms) [4] and if then recurrent symptoms develop, saccus revision surgery leads to successful reduction of symptoms in 80–90% of the cases [97], [98]. Intraoperatively, connective or granulation tissue is regularly found as well as newly formed bone around the area of the endolymphatic sac [99].

Among all surgical therapies that are mentioned in the context of MM, the best results regarding hearing preservation are achieved with interventions at the endolymphatic sac [100], [101] so that this kind of surgery can also be recommended for patients suffering from bilateral MM with success rates regarding vertigo control which can be compared to the one of unilaterally affected patients [102]. Beside a significant improvement of the specific symptoms, the quality of life can be significantly improved by interventions at the endolymphatic sac [101]. Before such an intervention, imaging should exclude a large vestibular aqueduct or tumors of the endolymphatic sac. Even patients older than 65 years do not have a higher perioperative morbidity for endolymphatic sac surgery [103] with a very low overall invasiveness so that saccus decompression is promoted from step III to II in our gradual scheme before gentamicin therapy in cases of usable hearing ability (Figure 1 (Fig. 1)). In all endolymphatic sac surgeries, the intraoperative application of corticosteroids is recommended for hearing preservation [27].

##### 2.2.1.4 Blockage of the endolymphatic duct

Saliba et al. described in 2015 a new, non-destructive surgical technique for treatment of MM where they perform a blockage of the endolymphatic duct. They compared this technique with endolymphatic sac decompression surgery in a prospective, non-blinded randomized trial [104]. After complete decompression of the endolymphatic sac, this intervention consists of exposing the endolymphatic duct medial to the saccus and ligating it with 2 titanium clips. In this way, the endolymphatic sac is isolated and the endolymph fluid in the inner ear is reduced so that the imbalance of the endolymph production and absorption is balanced by the blockage of the suspected overproduction of endolymph fluid in the saccus [105].

In the context of this study, 35 patients underwent blockage of the endolymphatic duct and 22 only saccus decompression. The postoperative results were assessed 1 week as well as 6, 12, 18, and 24 months afterwards. The recurrence of vertigo in the blockage group amounted to 3.5% after 6 and 24 months (1 patient), for the patients who had undergone decompression, it amounted to 80% after 6 months and 66% after 24 months. The symptoms of tinnitus and pressure sensation were significantly better in the blockage group after 24 months compared to the control group. With regard to the audiological results, there was no difference between both groups. In 14% of the patients who had undergone blockage of the endolymphatic duct, outflow of cerebrospinal fluid occurred intraoperatively during preparation of the dura, 11 of 35 patients presented the postoperative symptom of BPPV of the posterior semicircular canal; further important complications were not observed in neither of the groups. According to the study of Saliba et al., the new procedure of blocking the endolymphatic duct is clearly superior to saccus decompression, especially in terms of controlling vertigo symptoms. However, it is eye-catching that rather poor results are reported about patients who had undergone decompression (see also 2.2.1.3) with a recurrence rate of the vertigo symptoms in 80% after only 6 months. It also remains open why this study was not conducted in the (double) blind way in order to increase the scientific quality [106].

In summary, the endolymphatic duct blockage seems to be an interesting surgical technique that shows good vertigo control as well as a significantly increased quality of life after surgery [60]. However, in comparison to decompression of the endolymphatic sac, it is clearly more challenging regarding the performance and it is associated with a higher risk of intraoperative liquor leakage with similar results regarding vertigo control in comparison to the literature.

#### 2.2.2 Ablative and destructive procedures

**Preliminary remarks:** Among the non-surgical but ablative therapeutic procedures also the intratympanic gentamicin therapy must be mentioned with the risk of severe hearing loss as undesired side effect in some patients, independent from the duration or type of administration [107], however, it will not be in the focus of the following paragraphs.

##### 2.2.2.1 Endolymphatic/perilymphatic shunt: sacculotomy/cochleo-saccuolotomy/vestibulo-cochleo sacculotomy

In the 1960ies, a procedure was described that should lead to relief of the ELH by a perilymphatic/endolymphatic shunt [108], [109], [110], [111]. It was applied in patients with therapy-resistant vertigo after failure of alternative therapeutic options. The sacculus is punctured through the stapes footplate (according to Fick [109]) or through the round window membrane (according to Schuknecht [112]) and thus a permanent shunt is created, with accompanying destruction of the labyrinth and cochlear function. Earlier reports about the possibility to perform this intervention with just a low risk of postoperative hearing deterioration [111], [112] were probably partly due to the patient selection because further deterioration cannot be measured in cases of preoperative functional deafness or profound hearing loss [27]. In later studies conducted in patients with measurable residual hearing, those results could not be confirmed [113], [114]. Giddings et al. reported about sobering results after cochleo-sacculotomy with a significant hearing loss in 80% of the patients and recurrent vertigo attacks in an average follow-up period of 17 months in 4 of 11 patients so that again a destructive intervention had to be performed [114]. Wielinga et al. suggest sacculotomy and Kinney et al. recommend cochleo-sacculotomy because of the easy and less invasive surgery method especially for older patients as alternative to a labyrinthectomy. They describe a very good vertigo control, however, with significant hearing loss in nearly all patients [115], [116]. In a study that compares cochleo-sacculotomy and saccus decompression a significantly better vertigo control is reported in patients after cochleo-sacculotomy with significant hearing deterioration [117]. In this study, a selection bias was observed since the patients with preoperative impaired hearing rather undergo cochleo-sacculotomy instead of saccus decompression. Especially for older patients without usable residual hearing ability such a surgery with its low invasiveness may be indicated [118].

Cochleo-sacculotomy performed at the same time as cochlear implantation may be discussed for deaf MM patients with persisting vertigo symptoms. This procedure was described by Westhofen who reports very good results [27].

##### 2.2.2.2 Neurectomy of the vestibular nerve

In the 20^th^ century, a fluctuating interest could be observed regarding the performance of neurectomies of the VIII^th^ cranial nerve [119]. The early neurectomies performed already at the end of the 19^th^ and at the beginning of the 20^th^ century for the treatment of vertigo consisted of complete transection of the nerve via different access routes [119], [120]. Later, Dandy and McKenzie described the selective neurectomy of the vestibular nerve via a suboccipital approach aiming to separate the vestibular organ from the brain and thus achieve vertigo control [121], [122], [123]. After this, House renewed the interest in this surgery by introducing microsurgery via a transtemporal approach through the middle fossa [124]. Fisch modified this access route [125] and reported in a series of 100 cases that no patient at all had significant postoperative hearing loss [126]. Further modifications of the surgical technique and the approach were performed by Silverstein (retrolabyrinthine approach [127]) and Bremond (retrosigmoid approach [128], [129]). Silverstein reported about a combination of both approaches that vertigo attacks stopped completely in 85% of the cases and improved in further 7% with a significant hearing loss in only 4% [130]. Independent from the access, success rates of more than 90% are mentioned with meanwhile significantly lower mortality and reduced complication rates in comparison to interventions at the beginning of the 20^th^ century [131], [132], [133], [134], [135].

In a comparative study, Colletti et al. report about 209 patients who underwent neurectomy and 24 patients who received intratympanic gentamicin therapy (also called chemical labyrinthectomy). They found a vertigo control of 95.8% in the neurectomy group in comparison to 75% in the gentamicin group. The speech discrimination in the neurectomy group was reduced from 85 to 82% and in the gentamicin group from 87 to 65%. The gentamicin therapy is not a surgical intervention, however, it should be considered to be a destructive procedure. Patients have to be counseled about the relevant risk of hearing loss [3], [4]. In the early postoperative phase, neurectomy does not cause a relevant hearing deterioration in the vast majority of the cases. Comparing the long-term results of patient groups who had either undergone neurectomy or saccotomy or who were offered a surgical intervention that was refused, it becomes obvious, independent from the type of intervention, that in cases of poor hearing the situation is stabilized and that in cases of good preoperative hearing ability it deteriorates, which corresponds to the natural course of the disease in the group without surgical intervention [136]. Albera et al. report that meanwhile about 80% of patients with an indication of ablative therapy receive intratympanic gentamicin, the others are treated with neurectomy [35]. After neurectomy, insufficient vertigo control is reported to be about 5% and up to 20% in patients after gentamicin treatment [137].

The simultaneous [138] or sequential [139] combination of neurectomy with an endolymphatic sac surgery did not show advantages regarding vertigo control or hearing preservation compared to neurectomy of the vestibular nerve alone.

Setty et al. described a merely endoscopic retrosigmoid procedure and reported about improvement or completely stopped vertigo symptoms in more than 92% of 41 patients with a hearing preservation of 82.9% as well as a minimal craniotomy without retraction of the cerebellum [140].

**Annotation:** In the vast majority of the publications, neurectomy is reported although neurotomy is performed. Beside transection of the nerve fibers, neurectomy also includes an extirpation of Scarpa’s ganglion [59].

##### 2.2.2.3 Labyrinthectomy

For a long time, surgical labyrinthectomy has been considered as gold standard of surgical treatment for patients with therapy refractory MM when they did not have usable residual hearing or after failure of function-preserving surgical interventions [4], [141], [142] in which the residual hearing was sacrificed. Since Lake had described the first labyrinthectomy in 1904 [143], it took nearly to the middle of the 20^th^ century until labyrinthectomy could be established as less invasive alternative in comparison to neurectomy of the vestibular nerve [144], [145]. In further publications until nowadays, the surgical techniques reach from opening one semicircular canal and resecting the endolymphatic duct to the opening of all semicircular canals as well as the vestibulum with resection of the complete neuroepithelium, partly combined with the resection of Scarpa’s ganglion [146]. A comparison between labyrinthectomy and neurectomy as well as a combination of both does not show any superiority of one of the methods regarding vertigo control [147], [148], [149]. Especially for older patients, labyrinthectomy represents an alternative [150], [151], hereby the transmastoid approach should be preferred to the transmeatal access because of better vertigo control [151]. For a long time, the history of a labyrinthectomy was considered as relative contraindication for CI of MM patients despite the clear audiological criteria for this kind of hearing rehabilitation [152]. Temporal bone studies could not confirm the expected ossification and fibrosis of the cochlea after labyrinthectomy to the extent where an electrode could not be inserted in the scala tympani [153], [154]. Furthermore, MRI examinations of 18 patients with a condition after transmastoid labyrinthectomy revealed even 3 years after surgery a bilaterally equal T2-weighted signal intensity in the cochlea as hint for missing fibrosis or even ossification [142]. In contrast to this, fibrosis or ossification with the loss of the fluid signal in MRI examinations can be detected in one third of the patients who undergo a translabyrinthine access to the internal auditory meatus because of vestibular schwannoma. In those cases, the simultaneous cochlear implantation or for sequential surgery a previous dummy insertion is recommended [155]. For “simple” labyrinthectomy, this does not seem to be necessary. Deafness of MM patients caused by the disease itself is relatively rare [156]. In most of the cases, it is a consequence of different therapeutic methods so that enough spiral ganglia neurons for electrical stimulation should be available in the affected patients [153]. MM patients sometimes have even better results than other CI users [157]. More recent reports show very good results for labyrinthectomy simultaneously performed with cochlear implantation as well as for cochlear implantation performed sometime after labyrinthectomy (interval up to 21 years), so that CI can be recommended also in the patient group in cases of unilateral and bilateral affection [152], [158], [159], [160], [161].

Residual or recurrent symptoms regarding vertigo complaints are observed in up to 40% of patients after labyrinthectomy which is often explained by an incomplete destruction of the sensory epithelium and remaining circuits between the 1^st^ and 2^nd^ neuron of the vestibular pathway so that a resection of Scarpa’s ganglion should be performed [15].

Destructive surgical therapy modalities show the best success rates, however, there is a high risk for the complete loss of any existing residual hearing [162]. For ablative therapy procedures (labyrinthectomy and neurectomy) are success rates regarding vertigo control of 85–95% reported [4], [33], [163], [164]. In a study comparing both methods, no difference could be found with regard to the control of the vertigo attacks, however, a slight advantage regarding the postoperative instability sensation was found in the group of neurectomized patients [165]. This is very important for counseling towards or against an ablative intervention, but the estimation of the individual central compensatory possibilities have to be considered as well, especially because the respective patients get older and older [10]. Especially before considering destructive procedures, the risk of developing a bilateral MM must be discussed with the patient. Data in the literature are varying, the occurrence of Menière’s symptoms on the second side is reported in up to 78% of the cases [166], [167], [168], [169]. Whether the performance of function-preserving surgical procedures may delay or even avoid the occurrence of ELH or according symptoms, is still controversially discussed [102], [170].

Today, ablative surgical procedures are mainly justified after failed intratympanic gentamicin therapy [171].

Silverstein et al. compared the long-term course of Menière’s symptoms in a group of patients who had received surgical therapy (shunt surgery of the saccus or neurectomy) with the results of a group who had the recommendation of surgical therapy but refused [33]. It became obvious that in the non-surgery group after an average of 8.3 years no more vertigo attacks occurred in more than 70%. This has to be considered in the context of analyzing long-term results, but should not be given as justification to deny affected patients an early/direct success by surgical intervention. In order to reduce the influence of the natural course of the disease on the results that are due to a surgical intervention rather the one- and two-year success rates play a key role for the evaluation of different procedures [67].

Since the development of ELH is mostly multifactorial and probably rarely due to only one origin, no therapy method must be expected to be the standard for all patients. Moreover, different patient groups must be assessed systematically in a differentiated way regarding therapy success/failure [51]. In this way, future prognostic factors may be retrieved for the each method. 

## 3 Dehiscence syndromes

The dehiscence of the bone above the superior semicircular canal as cause of vertigo, oscillopsia, and specific types of nystagmus, sometimes accompanied by conductive hearing loss, pulsatile tinnitus, or autophonia as reaction on loud noise, intracranial pressure increase, or increased pressure in the middle ear, was first described by Minor in 1998 [172], [173]. The description of those pathological mechanisms is controversially discussed since then, including the whole disease as well as the diagnostic and the indication for surgical therapy [27]. The dehiscence syndrome of the superior semicircular canal was described shortly afterwards with the therapeutic recommendation of occlusion or resurfacing of the superior semicircular canal, both with an access through the middle fossa, based on the experience with 5 patients [174]. The pathophysiological explanation of dehiscence syndromes refers to 2 already known phenomena: the Tullio phenomenon (vertigo and nystagmus symptoms caused by noise [175], [176], [177]) and the Hennebert’s sign (syn: fistula sign; vertigo caused by pressure increase [178]). Meanwhile, also dehiscence syndromes have been described for the posterior and lateral semicircular canals as well as synchronous dehiscences of different semicircular canals [179], [180], [181], [182], [183]. The dehiscence of the lateral semicircular canal mostly occurs in relation with chronic otitis media (cholesteatoma) and is discussed in chapter 4 as labyrinth fistula. The theory of a mobile third window may well explain the different symptoms that many patients with semicircular canal dehiscence complain about [172], [184], [185], [186], [187], [188], [189]. Furthermore, animal models could well show the audiological consequences of the third window [190], [191], [192]. Critics of the dehiscence syndrome theory argue that in an investigation only slightly more than half of 65 patients with a dehiscence syndrome had conductive hearing loss [173]. Finally, the reason for developing a dehiscence syndrome remains unclear in the majority of the cases [193], [194].

Because of the high symptom variability in patients with dehiscence syndrome [195], [196], otosclerosis (conductive hearing loss with lost stapedius reflexes), tube disorders (hyper- and hypofunction), Menière’s disease, and perilymph fistula, are the most frequent differential diagnoses [197]. Especially patients who have persisting conductive hearing loss after middle ear intervention despite normal ossicular chain mobility should undergo the respective imaging to exclude a dehiscence syndrome. Already in 1980, House et al. created the term of inner ear conductive hearing loss after they had found no reflex at the round window membrane in patients with normal stapes mobility. The described “invisible inner ear pathology” was possibly a dehiscence of the superior semicircular canal so that already at that time the phenomenon of dehiscence syndrome could have been described [198]. Not all patients with the clinical and the radiological signs of a dehiscence syndrome need surgical therapy. In most of the cases, a satisfactory control of the disease can be achieved by avoiding the situations that cause the symptoms [174], [199], [200], [201], [202]. Only in about 10% of the patients with the constellation of complaints and findings of a dehiscence syndrome receive surgical therapy [27]. Vestibular symptoms are the most frequent reason for surgery [203]. When indicating surgery, the severity of the individual complaints and the associated impairment in the quality of life must be evaluated regarding the possible risks of surgery.

As surgical options, either the so-called resurfacing with cartilage, bone and/or fascia or as modification the covering with hydroxyl apatite or bone cement are possible. The last-mentioned procedure is also called “capping”. In both methods, the membranous semicircular canal is not closed or compressed but the continuity of the dehiscent bone is restored. In contrast, for occlusion (“plugging”) the according semicircular canal is compressed by muscle fascia, fibrous tissue, bone wax, or bone pâté and closed [204], [205], [206]. While the initial control, especially of the vestibular symptoms, seems to be more effective by resurfacing [207], [208], the recurrence of the symptoms after occlusion is lower [173], [209]. Possible access routes are craniotomy of the middle fossa as long-term transtemporal standard access [194], [209], [210], [211] as well as the transmastoid access that has gained in importance in the last years [196], [206], [212], [213], [214], [215], [216], [217] and also the complication-free access through the auditory canal with a round window covering is described [194]. For the last-mentioned procedure, the round window is closed with bone wax, muscle plugs or fascia [218]. Liming et al. described a navigation-based endoscopic 15 mm keyhole approach to the middle fossa after they had demonstrated the feasibility with temporal bone specimens [208]. An endoscopic procedure has also been suggested by other groups [194], [207], [219], [220].

Interventions at the middle fossa may lead to the development of epidural hematoma [221], [222], seizures [223], or liquor fistulas [224] as well as intracranial bleedings, brain edema or meningitis [225]. Schick et al. described a newly occurring temporal flap gliosis in 69% of the cases after a transtemporal approach to the middle fossa as well as conspicuities in neuropsychological tests one year after surgery [226]. A postoperative paresis of the facial nerve seems to occur more frequently in cases of dehiscence syndrome than in patients who undergo such an intervention for other reasons [222], [227]. Patients who had stapedoplasty because of conductive hearing loss have a higher risk of hearing deterioration after occlusion of the semicircular canal, independent from the approach [228]. 

The assessment of the current literature on the different approaches is rather difficult because the postoperative “success” is sometimes defined as improvement and sometimes as complete absence of the symptoms. Additionally, mostly small and inhomogeneous patient groups are described who had undergone different surgical techniques in individually modified ways. The success rates of the different surgical therapies reach from 75–100% for occlusion, resurfacing, capping or resurfacing and occlusion, independent from the access route [215], [216], [229], [230], [231]. For resurfacing, different materials are suggested (cartilage, fascia, squamous part of the temporal bone, mastoid skin). Ma et al. recommend a combination of temporal fascia and covering bone dust because this combination adapts to the individual surface properties the best [217]. Hahn et al., in contrast, use a hydroxyl apatite plate in a modified resurfacing technique [232].

While the transtemporal approach allows good visualization, orientation, and handling of the instruments [207], the transmastoid approach is a significantly less invasive access with the disadvantage of an unfavorable exposition of the bone defect [214], [233]. Generally, covering with bone cement or occlusion of the semicircular canal is also possible via a transmastoid approach [212], [233], [234], [235], [236]. After resurfacing with calotte bone of the squamous part, more frequently the preoperative symptoms reoccurred because of bone absorption [211], [214]. The bone thickness of the squamous part is mostly lower in patients with dehiscence syndrome compared to normal people so that often an absorption of the transplanted bone material with subsequent recurrent symptoms is observed [211]. In cases of extensive defects of the tegmen tympani and the mastoid that occur relatively often in patients with dehiscence syndrome, surgery with an individually designed Bioverit implant may be indicated after failure of classical resurfacing technique with cartilage and bone material [10].

In a meta-analysis comparing these surgical techniques (resurfacing, occlusion, capping) by Vlastarkos et al. [237] success rates of 50% for resurfacing which was significantly poorer than for occlusion (97%) or capping (93%) were reported. The most frequently occurring complication was sensory hearing loss and balance disorders. Another literature research showed better symptom control after occlusion in comparison to resurfacing, however, with a higher risk for postoperative deterioration of the bone conduction threshold [194], [238]. In contrast to the transtemporal approach, the transmastoid occlusion shows better results regarding the hearing ability [239] while the transtemporal technique reaches a hearing loss of up to 25%. Conversely, in a study of 24 interventions with temporal access, Goddard et al. reported about no significant hearing deterioration more than one year after surgery [240]. Powell et al. described a series of 20 surgically treated patients with dehiscence syndrome of the superior semicircular canal who experienced an improvement of the symptoms in 76% after transmastoid resurfacing. The patients without postoperative improvement were nearly exclusively those with atypical symptoms [241].

Comorbidities such as migraine often lead to prolonged hearing problems after surgical intervention of the semicircular canals [222]. Other diseases that mimic the symptoms of dehiscence syndrome should be examined by differential diagnostics and excluded if possible [227]. Regarding success rates and complications, a systematic review of Gioacchini et al. revealed no significant differences for 150 evaluated interventions regarding the access (middle fossa vs. transmastoid) or the surgery technique (resurfacing, capping, occlusion, or resurfacing with occlusion) with an overall success rate of 94% [230]. In an investigation of 84 interventions for dehiscence syndrome, Barber et al. found the development of BPPV mostly within 3 months after surgery, in the control group (patients without surgery) only in 6.2% [242]. The development of BPPV was independent from the different approaches or surgery techniques so that preoperative information of the patients should include this possibility.

Benamira et al. investigated which patients decide in favor or against surgical treatment. Patients with vestibular symptoms, hyperacusis for own footsteps and chewing noise, autophony, and stress-related oscillopsia decided more frequently for surgery [202].

Patients who mainly suffer from hyperacusis and do not have a positive Tullio phenomenon, may benefit from closure of the round window to stop their complaints [188]. In this way, the most easily accessible “third” window is closed: the round window. While the surgical risks are calculable, there is the risk that an enhanced movement of the fluid in direction of the dehiscent semicircular canal may trigger a postoperative Tullio phenomenon [188]. That is why this procedure should only be suggested to patients without Tullio phenomenon and be reversible by applying fascia, muscle or connective tissue [197], [218]. According to the current literature, a reinforcement or covering of the round window membrane in patients with dehiscence syndrome is generally possible for audiological and vestibular system control. The surgical risks are clearly less relevant than for alternative surgical procedures (resurfacing, occlusion, or capping), however, there are just a few experiences with this method and long-term results with adequate patient numbers are missing so far.

Patients who have bilateral dehiscences (radiologically confirmed in up to 50% [243], [244], [245]) should undergo surgery first of the side with the more severe symptoms. In most of the patients, symptoms stop after surgery so that surgery of the contralateral side is only rarely required. In cases of respective complaints, however, it may be performed with a positive outcome [231], [246], [247]. Agrawal et al. reported that after surgery of the first side the symptoms of the contralateral side are sometimes unmasked and that therefore the patients complain about different symptoms or deterioration of existing symptoms. The increased development of side effects in patients of this study is possibly due to the bilateral occlusion with subsequent oscillopsia [247], [248] so that resurfacing or capping should be preferred to occlusion for patients with bilateral dehiscence syndrome.

Studies on the quality of life after the first intervention could show a significant improvement for different surgical procedures of dehiscence syndromes [203], [249], [250]. For some symptoms, the success rate of up to 100% is mentioned, however, complete loss of the symptoms cannot be regularly achieved after the first intervention [227], [251].

With increasing diagnosis and surgical therapy of dehiscence syndromes in the last years, also the experience was enlarged regarding revision surgeries. Revisions are frequently required because of dislocated or absorbed resurfacing or occlusion material [246], [251]. Sharon et al. look back to 23 revision surgeries in 21 patients and report about nearly 2 third who experienced complete or at least partly elimination of the symptoms [251]. All patients underwent resurfacing with occlusion via a transtemporal approach in the context of revision intervention. There were no differences regarding the postoperative hearing outcome in comparison to patients after the first intervention, but the overall success rate was slightly lower. Especially in the context of revision, typical symptoms of dehiscence syndromes should be present and the patient should be intensively counseled before surgery about the lower success rate compared to the first intervention [251].

The diagnosis of dehiscence syndrome in children is difficult because often history taking, especially for vestibular symptoms, is not reliable. However, there is an increasing number of reports about the symptomatic occurrence of this disease even in children [252], [253], [254]. Children with dehiscence syndrome are often only because of audiological symptoms noticed so that the indication for surgical therapy is made very reluctantly [253], [255]. From the literature, the age group of children younger than 7 years has radiologically a clearly higher prevalence of dehiscence or very thin bone of the superior but also posterior semicircular canal, even if those data are variable [256], [257], [258]. Clinical symptoms in this age group are often not present [256]. Lee et al. presented 10 cases of pediatric dehiscence syndrome (average age: 6.9 years). One of those patients had conservative therapy for several years and after progression of the vestibular symptoms she underwent successful transtemporal surgery at the age of 14 years [253]. In children and adolescents, a surgical procedure is only performed in very distinct cases.

**Figure 3:**
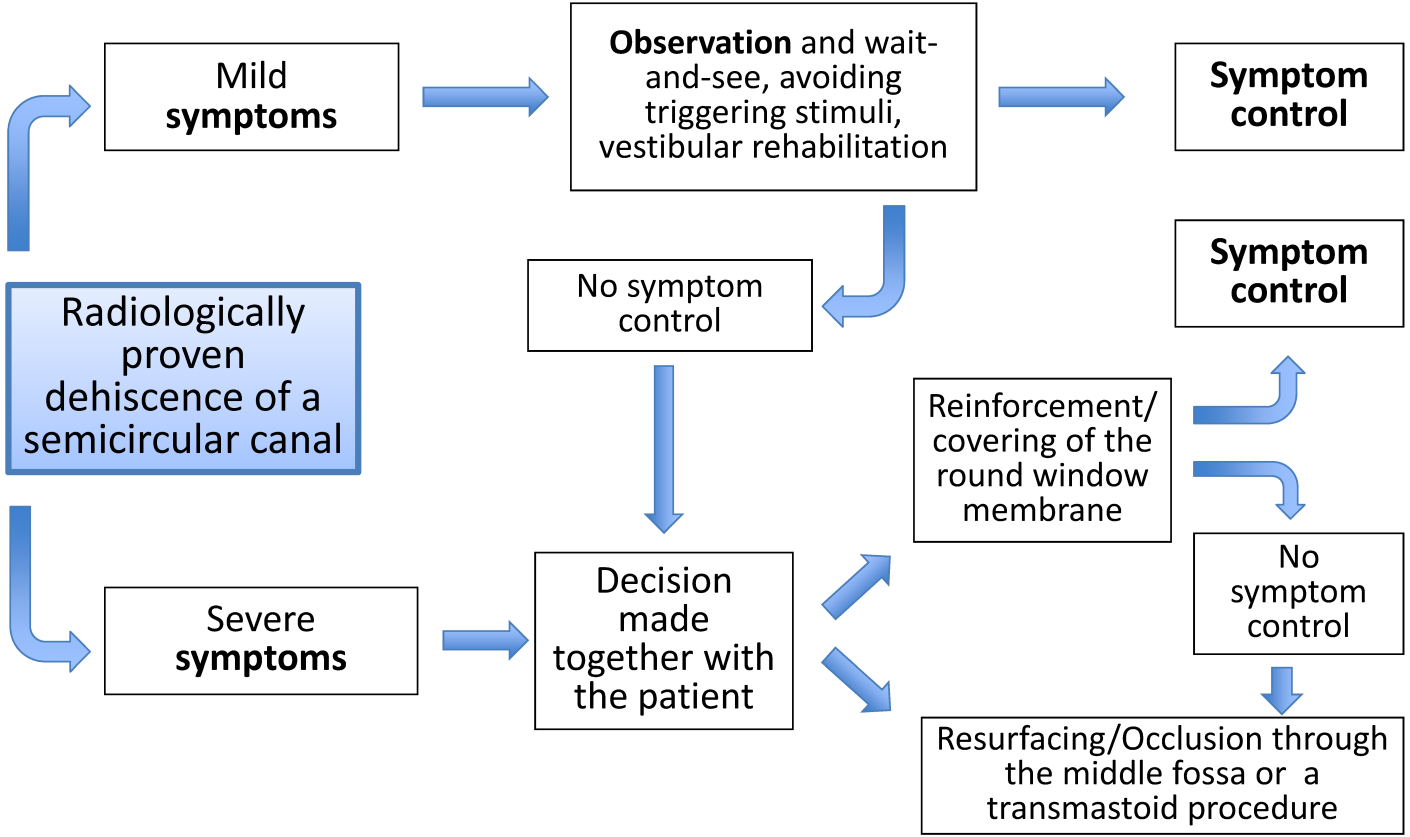
Therapeutic scheme for dehiscence syndrome of a semicircular canal (modified according to [197])

Dehiscence syndromes get more and more in the focus of otolaryngologists and physicians of other disciplines and reach an increasing differential diagnostic significance. The challenge of this disease consists of the clinical severity of very different cochleo-vestibular disorders and an appropriate patient selection for the various treatment procedures. For those patients who have disabling complaints which are impairing the quality of life the above-mentioned surgical therapies are available with good prognosis regarding symptom control (Figure 3 (Fig. 3)). Independent from the approach (transmastoid or transtemporal), the occlusion of the semicircular canal with simultaneous resurfacing of the dehiscent part seems to achieve the best results whereas the exact comparison of studies remains difficult.

## 4 Labyrinth/perilymph fistula

With exception of the round and the oval window, the membranous labyrinth is surrounded by the very dense petrous bone. Labyrinth fistulas are an unnatural connection between the inner ear and surrounding structures (middle ear, mastoid, dura, etc.) [259]. They include fistulas of the semicircular canals that might develop due to bone arrosion in the context of cholesteatoma, dehiscence syndromes of the semicircular canals (see chapter 3), and the so-called perilymph fistulas that are defined as perilymph leak through the round or oval window [260]. Similar to patients with ELH, there are also a multitude of sometimes confusing and contradictory classifications/definitions [260] that make systematic assessment of the therapeutic methods difficult because very different diseases have been compared with each other.

### 4.1 Perilymph fistula

Already in 1905, Hennebert noticed that patients with ear syphilis also suffer from vertigo or nystagmus caused by pressure changes (reduced or increased pressure) in the auditory meatus [178]. This so-called Hennebert’s sign was used as diagnostic test for ear syphilis, nowadays it is generally called fistula test (syn. fistula symptom). Positive or negative pressure in the auditory meatus, generated by a pneumatic otoscope, stimulates the respective receptors of the semicircular canals. Perilymph fistulas developed at the beginning of stapes surgery in the 1960ies because of larger prosthesis and the not performed sealing of the footplate region with connective tissue as it is standard nowadays [261], [262]. Perilymph fistulas were also described after cranial trauma, intracranial pressure increase, or without visible cause as spontaneous perilymph fistula [263], [264], [265]. Other possible reasons may be barotrauma after flying or diving, nose blowing, coughing, airbag traumas, but also acoustic traumas [260], [266], [267]. The symptoms of perilymph fistula are unspecific: vertigo, sensation of instability, staggering, sometimes accompanied with hearing loss [268]. Clear and specific diagnostic tests are missing so that the presence of a positive Hennebert’s sign or confirmed Tullio phenomenon sometimes led to generously indicating an explorative tympanoscopy in cases of suspected perilymph fistula. In the literature of the 1980ies and 1990ies, numerous studies are found about experiences with the surgical exploration because of suspected perilymph fistula [268], [269], [270], [271]. The data on intraoperatively confirmed perilymph leak vary between 40% and nearly 90% of all explored middle ears. The majority of the authors recommend occlusion of the round window niche and the footplate region with connective tissue, independent from the actual proof of perilymph leakage because even patients without intraoperative confirmation benefit in a similar positive way regarding the main symptoms compared to patients with confirmed diagnosis [268], [269], [272]. An explanation for this may be that some fistulas are only intermittently open [259]. While in the case of a fistula after cranial trauma nearly always the oval window was affected, leakages of the round window were found predominantly after barotrauma or unknown cause [260], [273]. Surgical techniques range from simple occlusion with connective tissue up to the application of a laser as well as the use of fibrin glue in order to reduce the re-occurrence of the symptoms after initially successful therapy from 27 to 8% [274].

Critical reviews and recent reports, partly also about endoscopic middle ear explorations for a suspected perilymph fistula, could not confirm the high rates of actually proven fistula mentioned in the literature and question in particular the existence of spontaneous perilymph fistulas because targeted history taking reveals trauma or other possibly triggering factors in most cases [260], [275], [276], [277]. Shea described in an impressive article from 1992 the myth of spontaneous perilymph fistula and doubted their existence because he had never seen such a phenomenon in more than 36,000 ear surgeries [278].

In our opinion and based on clinical experience, perilymph fistulas occur but they are due to direct trauma or barotrauma in most of the cases. If the suspicion and the according symptoms occur, tympanoscopy with occlusion of the round window niche and the footplate region is reasonable as therapeutic option after thorough counselling of the patient.

### 4.2 Arrosion of the semicircular canals by cholesteatoma

Cholesteatomas may cause a functional loss of the peripheral vestibular organ and/or perilymph fistula because of bone arrosion, that is mostly located at the horizontal semicircular canal, more rarely also at the round window or the promontory [259], [279]. In the context of urgent restoring surgery, the epithelium must be removed completely and the perilymph tube must be covered in several layers with connective tissue, bone dust, fibrin glue, cartilage, or specially designed Bioverit implants for larger defects [10], [279], [280]. Especially in patients with an open mastoid cavity and recurrent inflammation of the cavity, perilymph fistulas must be expected in revision surgeries [279]. Suspect areas should be explored at the end of surgery and in cases of a true fistula they should be covered immediately. Some authors recommend to leave the cholesteatoma matrix above the semicircular canal for large bone defects and to include it in the epithelization process of an open mastoid cavity, especially in the context of surgeries of the last hearing ear because of the risk of hearing deterioration/deafness [281], [282].

## 5 Benign peripheral paroxysmal positional vertigo

Benign paroxysmal positional vertigo (BPPV) is the most frequently observed type of vertigo with an origin in the inner ear especially in older patients [233], [283], [284]. Typically, rotational vertigo and horizontal-rotatory nystagmus occur in a crescendo-decrescendo course for 30–60 s after reclination of the head or body/head side position with a latency of a few seconds. More than 90% of the cases are idiopathic, fewer causes occur after trauma, after vestibular neuritis, or even after long-term bed rest [284], [285]. Even after surgical therapy due to dehiscence syndrome [230], otosclerosis [286], or even non-surgical interventions [284], BPPV is observed. The indication of surgery is only made in very rare cases [233], [287]. By means of different repositioning and positioning maneuvers, most cases can be treated successfully and permanently. If a therapy refractory BPPV is observed despite correct diagnostics and competently performed repositioning and positioning maneuvers, two surgical procedures may be a therapeutic option: partial neurectomy of the vestibular nerve (singular nerve) or occlusion of the affected (mainly posterior) semicircular canal [288]. 

### 5.1 Neurectomy of the singular nerve

In 1978, Silverstein suggested neurectomy of the singular nerve for the treatment of therapy refractory BPPV [289] which was and is nearly exclusively performed in North America [288]. Even for experienced surgeons, it is a technically challenging intervention and is associated with a significant risk of hearing deterioration as reported by Gacek in a study of 242 patients with 252 surgeries [290]. Beside Gacek who used an access through the auditory meatus only few other neuro-otologists have performed this intervention and published their results [291], [292], [293], [294], [295], [296], [297]. Silverstein described a postauricular approach with a maximum of drilling at the external auditory canal and reported about a complaint-free outcome of 80% in a series of 58 patients while just 3 patients had a significant postoperative hearing loss [291]. Various studies report a complete elimination of the symptoms in 75–100% of the cases. As possible reason for therapy failure, a false diagnosis, incomplete transection, failed identification of the nerve, or the presence of an accessory nerve portion are mentioned [288], [298]. Postoperative sensorineural hearing loss is described from 30 dB up to complete deafness and mainly depends on the surgeon’s experience. Gacek reports about 3.7% (252 neurectomies), Epley about 41% in a series of 12 patients [290], [292]. Anatomical examination of the position of the singular nerve performed by Leuwer et al. led to the conclusion that the nerve can be exposed in at least 14% up to more than 30% of the cases only by opening the basal cochlear turn. That is why they question strongly the excellent results regarding hearing preservation [299]. 

### 5.2 Occlusion of the posterior semicircular canal

In 1990, Parnes and McClure described the transmastoid occlusion of the posterior semicircular canal as a relatively easy therapeutic procedure without severe complications, first in patients who had also an advanced sensorineural hearing loss [300] and later also in normal hearing patients [301]. Beyea reported about 65 of those interventions that had been performed by the same surgeon and mentioned an elimination of the vestibular symptoms in all cases, however, 3 patients revealed a persisting inner ear hearing loss (2 patients had a mild and 1 patient a severe hearing loss, however, this last-mentioned patient had already undergone 2 previous interventions in the sense of neurectomy of the vestibular nerve) [233]. Meanwhile, this surgical technique is internationally established and the good results are reproducible [302], [303], [304], [305], [306], [307], [308]. In some cases, a low-grade, transient, high-frequency hearing loss is reported after the surgery [233], [304]. For further preservation of the hearing ability, some authors have suggested the application of a CO_2_ laser [309]. However, up to now no additional benefit could be confirmed [288].

Because of the very favorable results and experiences with occlusion of the posterior semicircular canal, this procedure is also suggested to patients who suffer from bilateral therapy refractory BPPV as sequential procedure. This shows the same results as in patients suffering on just one side from BPPV. The experience is rather low (n=6) but apart from the mentioned side effects (high-frequency hearing loss in one patient, transient postoperative balance disorders) no other severe complications occurred [233], [310]. Surgical interventions because of therapy refractory BPPV are only recommended for a highly selected group of patients and since the 1990ies this number is decreasing continuously [288]. This may be due to the improvement of diagnostic tools and the pathophysiological understanding of the diseases of the lateral and anterior semicircular canal as well as the respective repositioning maneuvers and also to the increasing number of diseases that are evaluated by differential diagnostics in the field of neurology that are very similar to the symptoms of BPPV [288]. Some authors recommend at least 12 months of conservative, physical (positioning and repositioning maneuvers) treatment with continuous verification of the correct diagnosis before surgical therapy may be offered [233], [288]. Patients who nonetheless need surgery for BPPV should undergo occlusion of the respective semicircular canal. 

## 6 Vestibular implants

For permanent uni- or bilateral functional loss of the peripheral vestibular organs, no further therapeutic option exists apart from physical therapy and the associated activation of central compensatory mechanisms. The quality of life of these affected patients is significantly impaired so that the interest is very high to provide a perspective of improving their situation [311]. Development of the first vestibular implants (VI) is expected to be very promising, also with regard to the great success of cochlear implants (CI) [312], [313], [314], [315]. After numerous and at least in parts very promising trials in animal models [316], [317], [318], [319], first VI were applied in humans, mainly in patients with bilateral loss of the vestibular function, but also in patients with MM [312], [314], [315], [320], [321]. Sensors of the VI register movements such as for rotation or acceleration (e.g. by means of gyroscope or accelerometer) and transform them into electrical impulses that stimulate the vestibular system via electrodes implanted in the semicircular canals near the respective ampulla. Depending on the stimulated semicircular canal, specific eye movements can be provoked in an animal model and also in humans which correspond to the vestibulo-ocular reflex (VOR) [315]. In rhesus monkeys, it was already possible to confirm that not only the VOR but also the orientation of the spatial position of the head can be influenced by electrical stimulation with VI [322]. Transferring the knowledge obtained in animal models to the application in humans was first rather difficult and led to some surprising and even disappointing results [315]. The reasons are manifold, for example other implants are used in humans than beforehand in the animal model. Because of the size of the target structure, there is a problem with the exact positioning of the stimulus electrode. The electrical fields and stimulus currents are still classified as very conservative in order not to cause undesired reactions such as pain or stimulation of the auditory system [315]. While the cochleo-vestibular functions in the animal model after implantation of the VI electrodes could be preserved [323], the few trials in humans show mainly other results [312]. Beside natural differences regarding the robustness of sensory structures of the hearing and vestibular organ of a single species, it must be taken into consideration that the implantations in animal models were performed in intact inner ears whereas the trials in humans were understandably performed only in patients with functional loss and thus damaged inner ears. Hence, the risk of further damage of the residual function of the vestibular organ as well as hearing function was clearly higher and the ability to recover from implantation trauma was reduced when a certain severity of previous damage was present [312]. The fact, however, that generally an intervention in/at the cochlea or a peripheral vestibular organ is possible without damaging the respective other part of the inner ear, is revealed by numerous cochlear implantations without damaging the vestibular organ and also by occlusion of the semicircular canal in the context of BPPV with hearing preservation (see chapter 5.2).

Reports about the development and first progress of vestibular implants recall the first reports about cochlear implantation. Hereby, the initial idea and objective were to facilitate lip-reading of the affected patients [324]. Those already optimistic expectations of the beginning were exceeded by far in the following decades [325]. If the development of vestibular implant systems may experience similar gain in knowledge and the same promising results as in cochlear implant technology had been shown during the last 40 years, will be seen in the future. 

## Conclusion

Most disease of the peripheral vestibular system can be treated successfully by means of conservative therapy methods. For patients who still suffer from defined vertigo symptoms after exhaustion of pharmaceutical as well as other conservative treatment and who are severely impaired regarding their quality of life, surgical procedures are often the only remaining alternative. Because of the sometimes difficult selection of the patients, the improved neuro-otological diagnostic tools, and especially the further development of intratympanic drug application (first of all steroids, gentamicin etc.), surgical procedures for disease of the peripheral vestibular organs continuously decrease. Before surgical therapy, the diagnosis and the side assignment have to be double-checked, a sufficiently long conservative attempt has to be performed, and the benefits and risks of the offered surgical intervention have to be individually discussed with the patient during counselling.

In general, function preserving procedures are preferred where available. For patients with Menière’s disease, in particular saccus surgery is an effective method with just minimal side effects that can also be repeated if needed. Hence, neurectomy as ultima ratio is only performed very rarely nowadays. After exploitation of conservative treatment in cases of benign paroxysmal positional vertigo, the occlusion of the respective semicircular canal turned out to be beneficial. This procedure can mostly be performed with hearing preservation.

The invasiveness of the applied method, i.e. the decision to perform a function preserving or ablative intervention, mostly depends on the amount of residual hearing and the individual vertigo-induced reduction of quality of life in each individual patient. This does not apply for suspected arrosion of a semicircular canal due to cholesteatoma or acute inflammatory disease of the mastoid. Here, the surgical covering/remediation has to be performed immediately and independent from the current hearing ability.

Regarding all revisions or interventions at the second, also affected side, the following aspects must be considered: repeated verification of the diagnosis, differential diagnostic exclusion also of central disease, and in particular in older patients the estimation of possible compensation mechanisms after any kind of destructive intervention.

## Abbreviations

BPPV – Benign paroxysmal positional vertigo

CI – Cochlear implant

DHI – Dizziness Handicap Inventory

ELH – Endolymphatic hydrops

MM – Menière’s disease

VI – Vestibular implant

VOR – Vestibulo-ocular reflex

## Notes

### Competing interests

The authors declare that they have no competing interests.
